# Actual and Perceived Knowledge About COVID-19: The Role of Information Behavior in Media

**DOI:** 10.3389/fpsyg.2021.778886

**Published:** 2021-12-15

**Authors:** Julia S. Granderath, Christina Sondermann, Andreas Martin, Martin Merkt

**Affiliations:** Deutsches Institut für Erwachsenenbildung, Bonn, Germany

**Keywords:** media effects, health information seeking, mass media, illusion of knowledge, COVID-19

## Abstract

The COVID-19 pandemic poses a health threat that has dominated media coverage. However, not much is known about individual media use to acquire knowledge about COVID-19. To address this open research question, this study investigated how the perceived threat is linked to media use and how media use is associated with perceived and actual knowledge about COVID-19. In a German online survey conducted between April 16 and April 27, 2020, *N* = 952 participants provided information on their perceived threat and media use to inform themselves about COVID-19. In this process, they indicated how well they were informed about COVID-19 (perceived knowledge) and subsequently completed a COVID-19 knowledge test (actual knowledge). Results indicated that individuals who felt more threatened by COVID-19 used media more often to inform themselves (*b* = 0.20, *p* < 0.001) but focused on fewer different media channels (*b* = 0.01, *p* < 0.001). Further, frequent media use was associated with higher perceived knowledge (*b* = 0.47, *p* < 0.001), but not with higher actual knowledge about COVID-19 (*b* = −0.01, *p* = 0.938), reflecting an illusion of knowledge. Additionally, using fewer media channels was linked to higher perceived (*b* = 2.21, *p* < 0.001) and actual knowledge (*b* = 2.08, *p* = 0.008). Finally, explorative analyses on the use of different media channels revealed that an illusion of knowledge emerged for using social media, public television, and newspapers. Potential explanations for the findings and implications for future research are discussed.

## Introduction

At the beginning of 2020, a novel coronavirus disease (COVID-19) has spread rapidly across the globe. As a response, the World Health Organization announced a pandemic outbreak in March 2020 (World Health Organization, 2020). With over 2 million cases when the data of this study were collected in April 2020 (Johns Hopkins University, 2020), the COVID-19 crisis has posed a global health threat, especially given that at this early stage of the pandemic, not much was known about the transmission, the symptoms, and the long-term effects of the potentially deadly disease caused by the virus. Since its outbreak, COVID-19 has dominated media coverage worldwide, with the media playing a central role in communicating information about the COVID-19 pandemic to the public. Given that knowledge about the pandemic can be considered an important foundation for the successful containment of COVID-19 (Algara et al., 2020), it is essential to understand how individuals use media to stay informed and how they acquire knowledge about the COVID-19 pandemic through media.

Although increased information seeking in media has been well documented to occur during times of crises (e.g., Spence et al., 2005; Lachlan et al., 2009), there are no studies to date that systematically investigated how different patterns of media use during crises are linked to actual and perceived knowledge about the crisis. The present study tackles this research gap by focusing on the associations between participants’ perceived threat, patterns of media use, i.e., the intensity of media exposure (media exposure henceforth) and variety of media channels exposed to (media width henceforth), and their actual and perceived knowledge about COVID-19. Subsequently, we will first describe how perceived threat during a pandemic may affect people’s information-seeking behavior in the media. Following this, we will briefly outline how the increased use of media to stay informed about the pandemic may be associated with people’s actual and perceived knowledge about COVID-19.

### Media Use in Times of Crises

Over the last decades, individual media use, also in times of crises, has been extensively researched, providing a broad body of theories and empirical studies. Since crises lead to high levels of uncertainty (Seeger et al., 1998), the majority of studies in the field builds on the uncertainty reduction theory (URT) proposed by Berger and Calabrese (1975). According to URT, individuals react to high levels of uncertainty with increased information-seeking behavior as a coping strategy. In doing so, individuals aim at (1) increasing the predictability of a situation and (2) reducing the aversive state of uncertainty (Heath and Gay, 1997). Especially when uncertainty is linked to a perceived threat (e.g., a potentially harmful outcome), the need for information increases (Heath and Gay, 1997; Brashers et al., 2000). Focusing on the context of crises, information-seeking behavior using media has already been well documented in various contexts (Murch, 1971; Heath et al., 1995): Scholars observed information-seeking behavior in response to terror attacks (Spence et al., 2005, 2006; Lachlan et al., 2009), natural disasters (Burke et al., 2010; Rahmi et al., 2019), and different health issues (Brashers et al., 2000; Lu, 2003; Walter et al., 2012; Zhao and Zhang, 2017).

In line with this, the media system dependency theory (Ball-Rokeach and DeFleur, 1976) predicts higher media needs in times of societal changes and conflicts. Hence, according to Ball-Rokeach (1985), the perception of threat (e.g., psychological, economic, physical) leads to stronger media dependencies, i.e., the extent to which individuals depend on the media as a resource to interpret and organize information on potentially threatening situations such as crises. This relation between perceived threat and need for information became evident in different studies (e.g., Loges, 1994; Lowrey, 2004). However, whereas these studies addressed the need for information, they did not investigate how the use of different media channels is associated with both individual’s actual and perceived knowledge.

### (Perceived) Knowledge Acquisition Through Media Use

Based on the research presented above, it can be stated that perceived threat is associated with an increased tendency to seek relevant information, and along with this, with increased exposure to corresponding information in the media. Research (e.g., Barabas and Jerit, 2009) already showed that mass media provide information that may affect one’s knowledge. However, the relationship between media exposure and knowledge remains unclear: While some studies suggested a positive association between media exposure and actual knowledge on specific topics (De Vreese and Boomgaarden, 2006; Kaufhold et al., 2010; Marquart et al., 2019), other studies showed that this association only holds true for specific media channels (Drew and Weaver, 2006; Wei and Lo, 2008; Dimitrova et al., 2014). Beyond media exposure, which was mostly measured by the individual frequency of media use, other studies (e.g., Stamm et al., 2000; Taddicken and Neverla, 2011) also investigated the width of media use by including the number of used media channels in their analyses. These studies found positive associations between media width and actual knowledge, suggesting that wide media use may increase the chances of exposure (see Barabas and Jerit, 2009).

However, although exposure to information may be considered a necessary precondition for acquiring knowledge about an issue, it is not sufficient. In particular, while some studies observed positive associations between media exposure and actual knowledge (De Vreese and Boomgaarden, 2006; Kaufhold et al., 2010; Marquart et al., 2019), there also is growing evidence suggesting that increased exposure to media may be associated with an increase of participants’ perceived knowledge (Zhao, 2009), which is not necessarily reflected in actual gains in knowledge, resulting in an illusion of knowledge (Leonhard et al., 2020; Schäfer, 2020).

Illusions of knowledge are defined as a discrepancy between perceived knowledge and actual knowledge or actual ability to perform a task, resulting in overconfidence (Park, 2001; Avhustiuk et al., 2018; Kardas and O’Brien, 2018; Schäfer, 2020) that is “likely to retard learning” (Epstein et al., 1984, p. 355). This overconfidence may be induced by perceived easiness (Salomon, 1984; Scharrer et al., 2017), fluency (Toftness et al., 2018), or familiarity (Metcalfe et al., 1993) of the provided information. While these factors depend on the specific characteristics of the information (e.g., fluency of the speaker, Toftness et al., 2018), they may also be induced by repeated exposure to information (Bornstein and D’Agostino, 1994; Koriat, 1995, 2000). In line with this reasoning, Kardas and O’Brien (2018) demonstrated that repeated exposure to a video resulted in participants’ overconfidence in their ability to master the task.

Such selective effects of increased exposure to information on perceived knowledge, but not on actual knowledge, were also observed in two recent studies investigating the associations between participants’ use of online news sources and their actual and perceived knowledge (Leonhard et al., 2020; Schäfer, 2020). In an online experiment, Schäfer (2020) manipulated participants’ news feed ranging from a high amount of news posts to no news posts, and compared factual and perceived knowledge between the different news feed conditions: Whereas participants exposed to a news feed with a high amount of news posts reported higher perceived knowledge scores compared to the control group without news posts in their news feed, there were no differences in factual knowledge between these conditions. Relatedly, an online survey by Leonhard et al. (2020) identified different online news usage patterns and examined associations of these usage patterns with actual and perceived political knowledge. For some patterns characterized by a high frequency of online news usage, the authors observed an illusion of knowledge, with participants overestimating their own political knowledge.

While the study of Schäfer (2020) relied on an experimental approach focussing on a specific topic, the study of Leonhard et al. (2020) chose a broader approach covering political knowledge in general, relying on participants’ self-reports on their actual media use. In the current study, we intended to combine the strengths of the different approaches by investigating the associations between participants’ self-reported media use, actual knowledge, and perceived knowledge on a specific topic in the context of the COVID-19 crisis as a dynamic, ongoing pandemic situation. This targeted approach limited to one specific topic (i.e., COVID-19) allowed us to better relate the participants’ actual knowledge to their news consumption about COVID-19, instead of relying on more global assessments that may result in an underestimation of the relationship of news consumption and the measured actual knowledge.

## The Present Research

Previous research made important contributions to the literature on information behavior during crises and the effect of media use on knowledge. However, none of the reported studies systematically investigated the associations between perceived threat and different parameters of media use (i.e., media exposure and media width) and systematic associations between these parameters and actual and perceived knowledge. The COVID-19 pandemic constitutes a real-world scenario in which these research gaps can be addressed. In particular, it is a unique situation characterized by (1) increased levels of potential threat, (2) intense media coverage of the pandemic, and (3) emerging knowledge that needs to be transmitted to the public.

Different theories and prior research suggest an association of perceived threat with an increased need for information (e.g., Ball-Rokeach and DeFleur, 1976; Ball-Rokeach, 1985; Loges, 1994; Lowrey, 2004) and with more intense information seeking in media (e.g., Berger and Calabrese, 1975; Heath and Gay, 1997; Spence et al., 2005; Lachlan et al., 2009). Based on this, we expected to find this association for information behavior during the COVID-19 pandemic.

*(H1) Threat hypothesis:* The higher the perceived threat of COVID-19, the higher is the individual media use (i.e., media exposure, media width) to inform oneself about the pandemic.

Research could further demonstrate that one’s media use is often positively associated with one’s knowledge: The higher the media use (i.e., media exposure, media width), the higher the knowledge about highly relevant issues, such as political and scientific topics (e.g., Stamm et al., 2000; Kaufhold et al., 2010; Taddicken and Neverla, 2011). Thus, based on the reported studies, it is feasible that the media use during the COVID-19 pandemic is also positively associated with one’s pandemic-related knowledge:

*(H2) Actual knowledge hypothesis:* The higher one’s media use to inform oneself about COVID-19 (i.e., media exposure and media width), the higher is the actual knowledge about COVID-19.

Previous studies further revealed associations between media use and perceived knowledge (Zhao, 2009; Schäfer, 2020). Therefore, we expected positive associations between media use and perceived knowledge:

*(H3) Perceived knowledge hypothesis*: The higher an individual’s media use to inform oneself about COVID-19 (i.e., media exposure and media width), the higher is the perceived knowledge about COVID-19.

Even though previous research implies positive associations of media use with both actual (H2) and perceived knowledge (H3), there still may be discrepancies between actual and perceived knowledge, reflecting an illusion of knowledge (Leonhard et al., 2020; Schäfer, 2020). Hence, we explored whether illusions of knowledge emerged and whether these illusions of knowledge became evident for different media channels. Further, we explored whether the different media use parameters mediate the relationship between perceived threat and knowledge parameters by conducting mediation analyses.

## Materials and Methods

### Participants

A total of *N* = 1,307 German residents filled out the online questionnaire during the first national lockdown in Germany (field time: April 16th, 2020–April 27th, 2020). Based on quota sampling, participants were recruited *via* the online panel provider Lucid, ensuring that our sample corresponded to the distribution of different demographic characteristics (gender, age, federal state) in Germany (see Table 1). They received financial compensation. We excluded “speeders” whose completion time was more than 30% below the median completion time, resulting in a sample of *N* = 1,053. We further excluded participants that showed implausible response behavior within the questionnaire.^1^ Further, we excluded 17 participants that did not provide any information on their migration background and three participants that indicated their gender as ‘diverse’, since we could not include them in the later analyses in which we needed these individual characteristics to match the participants (group size too small for matching). This resulted in a final sample size of *N* = 952 participants (487 female, 465 male, *M*_age_ = 49.46 years, *SD_age_* = 17.12) out of which 39.60% (*n* = 377) lived in a rather rural area and 60.40% (*n* = 575) lived in a rather urban area. Regarding education, 63.45% (*n* = 604) had vocational training, 20.69% (*n* = 197) had a university degree and 15.86% (*n* = 151) had no vocational training. Further, 55.04% (*n* = 524) were employed, 5.36% (*n* = 51) unemployed, and 39.60% (*n* = 377) inactive (e.g., pensioners). In addition, 88.87% of the participants (*n* = 846) had no migration background, 6.20% (*n* = 59) had a first-generation migration background, 3.36% (*n* = 32) had a second-generation migration background and 1.58% (*n* = 15) had a third-generation migration background.

**Table 1:** Distribution of gender, age, and federal states in the study sample and Germany.

	Study sample (*N* = 952)		Distribution in Germany (based on Destatis)	
	Total	%	Total	%
Gender				
Male	465	48.84	40,966,691	49.35
Female	487	51.16	42,052,522	50.65
Age				
18–24	97	10.19	6,304,136	9.08
25–34	134	14.08	10,602,364	15.27
35–44	131	13.76	10,079,154	14.52
45–54	160	16.81	12,460,467	17.95
55–64	170	17.86	12,092,132	17.42
65+	260	27.31	17,883,532	25.76
Federal state				
Baden-Württemberg	121	12.71	11,069,533	13.33
Bavaria	146	15.34	13,076,721	15.75
Berlin	45	4.73	3,644,826	4.39
Brandenburg	33	3.47	2,511,917	3.03
Bremen	7	0.74	682,986	0.82
Hamburg	20	2.10	1,841,179	2.22
Hesse	71	7.46	6,265,809	7.55
Lower Saxony	87	9.14	7,982,448	9.62
Mecklenburg West Pomerania	20	2.10	1,609,675	1.94
North Rhine-Westphalia	205	21.53	17,932,651	21.60
Rhineland-Palatinate	44	4.62	4,084,844	4.92
Saarland	12	1.26	990,509	1.19
Saxony	54	5.67	4,077,937	4.91
Saxony-Anhalt	28	2.94	2,208,321	2.66
Schleswig-Holstein	35	3.68	2,896,712	3.49
Thuringia	24	2.52	2,143,145	2.58

### Measures

#### Media Use – Media Exposure and Media Width

We investigated individuals’ media exposure and width of media use. For this purpose, participants were asked to indicate how often they currently use different media channels to inform themselves about the COVID-19 pandemic. In particular, participants indicated their use of public television, private television, newspapers, online news, radio, social media, and podcasts (e.g., “How often do you currently use the media channel ‘radio’ to inform yourself about the coronavirus^2^?”) on seven-point Likert scales ranging from “*never*” (1) to “*very often*” (7). Participants’ overall media exposure was determined by averaging the individual scores for the seven media channels. Hence, individuals’ potential scores ranged between 1 and 7.

The width of media use describes whether individuals’ information behavior is concentrated on one media channel or distributed across different media channels. To determine the width of media use, we calculated standardized Lorenz coefficients as a measure of concentration derived from the Lorenz curve. The Lorenz coefficient was developed to allow the comparison of distributions of a value (Gastwirth, 1971; Hartung et al., 2009). A coefficient close to 1 indicated a low media width, hence a one-sided media use. A coefficient close to 0 indicated a high media width, hence a broad media use.

#### Perceived Threat

We asked the participants to assess the individual threat posed by the COVID-19 pandemic (“How strongly do you perceive the coronavirus as a threat?”) on a seven-point Likert scale from “*a very low threat*” (1) to “*a very serious threat*” (7).

#### Perceived Knowledge

The participants indicated their perceived knowledge about the COVID-19 pandemic (“How well informed are you about the coronavirus?”) on a seven-point Likert scale from “*very poor*” (1) to “*very good*” (7).

#### Actual Knowledge

The actual knowledge about COVID-19 was assessed with a self-constructed knowledge test consisting of 26 true/false statements on the COVID-19 pandemic (see Supplementary Table A.1 in the online appendix). We limited the processing time of each statement to a maximum of 30 s in order to prevent the participants from looking up the correct answers online. We developed the 26 statements based on the information given on the website of the German Robert-Koch-Institute, which is the government’s central scientific institution in the field of biomedicine (website status from 14th April 2020). The statements covered various aspects of COVID-19, e.g., the correct name of the virus and the virus-induced disease, the transmission and its prevention, and virology terms (e.g., reproduction rate). The points for each correctly answered item were summed up to an overall score for every participant, so that the participants could achieve a maximum of 26 points in the knowledge test. Cronbach’s alpha for the actual knowledge test was *α* = 0.69.

### Procedure

We conducted an online survey.^3^ Informed consent was obtained from all individual participants. First, participants indicated their media use and the trustworthiness of different media channels. After that, we specifically asked about the use of podcasts in general, as well as the use of several specific COVID-19-related podcasts (this latter data is not included in this manuscript). Then, participants were asked how well they are informed about the COVID-19 pandemic (perceived knowledge). After that, they answered 26 knowledge questions (true/false) about the COVID-19 pandemic (actual knowledge). In the end, they answered different demographic questions and different questions on COVID-19 (e.g., on their personal experiences with COVID-19, the perceived threat of COVID-19). Overall, the survey took about 15 min.

### Analytical Strategy

We applied different statistical approaches that account for the limitations arising from our cross-sectional study design. We complemented multiple linear regression analyses and additionally conducted more restrictive propensity score analyses (i.e., dose response functions) that allow to reduce biases in non-randomized samples (Rosenbaum and Rubin, 1983). Specifically, to test the threat hypothesis (H1), the actual knowledge hypothesis (H2), and the perceived knowledge hypothesis (H3), we constructed generalized propensity scores^4^ (GPS, Hirano and Imbens, 2004). By constructing GPS, we controlled for different covariates on which the GPS estimation is based (Bia and Mattei, 2008). These variables entered in our models were age, gender, migration background, education, employment status, and living area (urban/rural). Based on the GPS, dose–response functions were estimated by including the GPS as an interaction effect with the treatment in a multiple linear regression equation. This procedure allows to account for biases caused by differences in the covariates (Hirano and Imbens, 2004). When estimating the dose–response functions, we additionally controlled for other relevant covariates (e.g., media exposure or media width to account for the structural dependence between these two media use parameters). The same covariates were also included in the corresponding multiple linear regression analyses.

Statistical analyses were performed with Stata and SPSS. Scripts and the online appendix can be downloaded at OSF.^5^

## Results

Table 2 shows means and standard deviations of perceived threat, media exposure, media width, actual knowledge, and perceived knowledge, as well as the correlations of the variables.

**Table 2:** Means, standard deviations and correlations (N = 952).

	Descriptives		Correlations				
	*M*	*SD*	1.	2.	3.	4.	
1. Perceived threat^a^	4.85	1.59				
2. Media exposure^a^	3.79	1.19	0.299^***^			
3. Media width^b^	0.32	0.12	−0.063	−0.545^***^		
4. Perceived knowledge^a^	5.43	1.21	0.194^***^	0.366^***^	−0.029	
5. Actual knowledge^c^	15.06	2.37	0.002	−0.044	0.095^**^	0.067^*^

^*^p < 0.05, two-tailed. ^**^p < 0.01, two-tailed. ^***^p < 0.001, two-tailed.

a *Perceived threat, media exposure, and perceived knowledge were answered on a 7-point Likert scale ranging from 1 to 7*.

b *The value of media width varies between 0 (broad media use) and 1 (one-sided media use)*.

c *For actual knowledge, the minimum score was 0 and the maximum score was 26*.

### Threat Hypothesis

To test the threat hypothesis on the association between perceived threat and media use to inform oneself about COVID-19, we performed regression analyses and dose–response functions separately for media exposure and media width. Regarding media exposure, the significant regression equation referring to the entire model, *F*(13,938) = 47.61, *p* < 0.001, *R*^2^ = 0.398, is displayed in Table 3a. The results showed that perceived threat was positively associated with media exposure, *b* = 0.20, *p* < 0.001, while controlling for covariates. The additional variance explained by the predictor media exposure was *ΔR*^2^ = 0.067.^6^ Furthermore, we performed the more restrictive analysis with the dose–response function based on the GPS for perceived threat. Due to the small sample size of participants with migration background, there is moderate evidence against the balancing property. Nevertheless, the balancing property of the GPS was fulfilled at a level of *p* = 0.05. The dose–response function also revealed a positive association between perceived threat and media exposure, *b* = 0.23, *p* < 0.001. Increasing levels of perceived threat were linked to increasing levels of media exposure.

**Table 3A:** Regression of media exposure on perceived threat.

Variable	*B*	*β*	*SE*	*t*	*p*	95% *CI*
Perceived threat						
Perceived threat	0.20	0.262	0.02	10.21	<0.001	[0.16, 0.23]
Gender						
Male (ref.: female)	−0.15	−0.063	0.06	−2.44	0.015	[−0.27, −0.03]
Age group^a^						
2 (ref.: group 1)	0.24	0.071	0.11	2.26	0.024	[0.03, 0.45]
3 (ref.: group 1)	0.34	0.098	0.11	3.08	0.002	[0.12, 0.55]
4 (ref.: group 1)	0.29	0.098	0.10	2.86	0.004	[0.09, 0.49]
5 (ref.: group 1)	0.36	0.131	0.11	3.37	0.001	[0.15, 0.57]
6 (ref. group 1)	0.64	0.149	0.14	4.48	<0.001	[0.36, 0.92]
Migration background						
Migration background (ref.: no migration background)	0.10	0.027	0.10	1.02	0.310	[−0.09, 0.30]
Education						
Vocational training (ref.: no vocational training)	0.22	0.089	0.09	2.38	0.018	[0.04, 0.40]
University degree (ref.: no vocational training)	0.33	0.112	0.11	3.02	0.003	[0.11, 0.54]
Employment status						
Employed (ref.: unemployed)	0.08	0.033	0.08	1.03	0.303	[−0.07, 0.23]
Area						
Urban (ref.: rural)	−0.05	−0.021	0.06	−0.80	0.425	[−0.17, 0.07]
Media width^b^						
Media width^b^	−5.28	−0.546	0.26	−20.62	<0.001	[−5.79, −4.78]
Constant						
Constant	4.08		0.17	24.64	<0.001	[3.76, 4.41]

*Ref. = reference group*.

a *Age groups: group 1 (18–30), group 2 (31–40), group 3 (41–50), group 4 (51–60), group 5 (61–70), group 6 (>70)*.

b *The value of media width varies between 0 (broad media use) and 1 (one-sided media use)*.

Regarding media width, the significant regression equation referring to the entire model, controlling for covariates, *F*(13,938) = 42.24, *p* < 0.001, *R*^2^ = 0.369, is displayed in Table 3b. The results revealed a positive association between perceived threat and the standardized Lorenz coefficient, *b* = 0.01, *p* < 0.001, when all covariates were entered in the model. The additional variance explained by the predictor media width was *ΔR*^2^ = 0.010. Hence, higher levels of perceived threat were related to a higher standardized Lorenz coefficient (i.e., a smaller media width). The more restrictive dose–response function, including GPS for perceived threat, showed similar results: Perceived threat predicted higher standardized Lorenz coefficients, *b* = 0.01, *p* = 0.012. A higher perceived threat was linked to a higher standardized Lorenz coefficient and thus a smaller media width.

**Table 3B:** Regression of media width^b^ on perceived threat.

Variable	*B*	*β*	*SE*	*t*	*p*	95% *CI*
Perceived threat						
Perceived threat	0.01	0.106	0.00	3.86	<0.001	[0.00, −0.01]
Gender						
Male (ref.: female)	−0.01	−0.056	0.01	−2.12	0.035	[−0.03, −0.00]
Age group^a^						
2 (ref.: group 1)	0.02	0.054	0.01	1.68	0.094	[−0.00, 0.04]
3 (ref.: group 1)	0.04	0.116	0.01	3.58	<0.001	[0.02, 0.06]
4 (ref.: group 1)	0.06	0.189	0.01	5.41	<0.001	[0.04, 0.08]
5 (ref.: group 1)	0.06	0.215	0.01	5.49	<0.001	[0.04, 0.08]
6 (ref.: group 1)	0.09	0.197	0.02	5.82	<0.001	[0.06, 0.12]
Migration background						
Migration background (ref.: no migration background)	−0.00	−0.003	0.01	−0.12	0.906	[−0.02, 0.02]
Education						
Vocational training (ref.: no vocational training)	−0.01	−0.031	0.01	−0.82	0.412	[−0.03, 0.01]
University degree (ref.: no vocational training)	0.00	0.011	0.01	0.28	0.779	[−0.02, 0.03]
Employment status						
Employed (ref.: unemployed)	−0.01	−0.043	0.01	−1.33	0.184	[−0.03, 0.01]
Area						
Urban (ref.: rural)	−0.01	−0.038	0.01	−1.46	0.146	[−0.02, 0.00]
Media exposure						
Media exposure	−0.06	−0.572	0.00	−20.62	<0.001	[−0.06, −0.05]
Constant						
Constant	0.48		0.02	30.09	<0.001	[0.45, 0.51]

*Ref. = reference group*.

a *Age groups: group 1 (18–30), group 2 (31–40), group 3 (41–50), group 4 (51–60), group 5 (61–70), group 6 (>70)*.

b *The value of media width varies between 0 (broad media use) and 1 (one-sided media use)*.

### Actual Knowledge Hypothesis

To test the actual knowledge hypothesis on the association between media use and actual knowledge about COVID-19, we conducted regression analyses and dose–response functions separately for both media exposure and media width.

For the regression of actual knowledge on media exposure, the regression equation referring to the entire model, controlling for all covariates (see Table 4a), was significant, *F*(14,937) = 2.41, *p* = 0.003, *R*^2^ = 0.035. However, regarding the association between media exposure and actual knowledge about COVID-19, both the regression analysis, *b* = −0.01, *p* = 0.938, and the dose response function based on the GPS for media exposure, *b* = −0.15, *p* = 0.171, found no significant effect. In line with this, there was no additional variance explained by the predictor media exposure in the regression analysis (*ΔR*^2^ = 0.000).

**Table 4A:** Regression of actual knowledge on media exposure.

Variable	*B*	*β*	*SE*	*t*	*p*	95% *CI*
Media exposure						
Media exposure	−0.01	−0.003	0.08	−0.08	0.938	[−0.17, 0.16]
Perceived threat						
Perceived threat	0.03	0.017	0.05	0.49	0.622	[−0.08, 0.13]
Gender						
Male (ref.: female)	0.07	0.015	0.15	0.46	0.647	[−0.23, 0.37]
Age group^a^						
2 (ref.: group 1)	0.00	0.000	0.27	0.01	0.996	[−0.53, 0.53]
3 (ref.: group 1)	0.35	0.051	0.28	1.26	0.209	[−0.20, 0.89]
4 (ref.: group 1)	−0.06	−0.011	0.26	−0.25	0.804	[−0.57, 0.44]
5 (ref.: group 1)	0.01	0.002	0.27	0.05	0.960	[−0.52, 0.55]
6 (ref.: group 1)	−0.03	−0.004	0.37	−0.09	0.929	[−0.75, 0.69]
Migration background						
Migration background (ref.: no migration background)	−0.04	−0.006	0.25	−0.17	0.863	[−0.54, 0.45]
Education						
Vocational training (ref.: no vocational training)	0.06	0.012	0.23	0.26	0.793	[−0.40, 0.52]
University degree (ref.: no vocational training)	0.90	0.153	0.28	3.25	0.001	[0.35, 1.43]
Employment status						
Employed (ref.: unemployed)	0.19	0.041	0.19	1.02	0.310	[−0.18, 0.57]
Area						
Urban (ref.: rural)	−0.19	−0.039	0.16	−1.19	0.233	[−0.50, 0.12]
Media width^b^						
Media width^b^	2.04	0.105	0.78	2.61	0.009	[0.50, 3.57]
Constant						
Constant	14.03		0.54	26.10	<0.001	[12.98, 15.09]

*Ref. = reference group*.

a *Age groups: group 1 (18–30), group 2 (31–40), group 3 (41–50), group 4 (51–60), group 5 (61–70), group 6 (>70)*.

b *The value of media width varies between 0 (broad media use) and 1 (one-sided media use)*.

Regarding width of media use, the significant regression equation referring to the entire model, *F*(10,941) = 3.11, *p* < 0.001, *R*^2^ = 0.032, is displayed in Table 4b. We found that when controlling for all covariates, the standardized Lorenz coefficient was positively linked to actual knowledge about COVID-19, *b* = 2.08, *p* = 0.008. The additional variance explained by the predictor media width was *ΔR*^2^ = 0.007. The more restrictive dose–response function supported this finding. Contrary to our Hypothesis 2, a lower width of media use (i.e., a higher Lorenz coefficient) was associated with higher actual knowledge about COVID-19, *b* = 3.33, *p* = 0.010.

**Table 4B:** Regression of actual knowledge on media width.

Variable	*B*	*β*	*SE*	*t*	*p*	95% *CI*
Media width^b^						
Media width^b^	2.08	0.108	0.78	2.67	0.008	[0.55, 3.61]
Perceived threat						
Perceived threat	0.03	0.018	0.05	0.52	0.601	[−0.07, 0.13]
Gender						
Male (ref.: female)	0.06	0.014	0.15	0.42	0.676	[−0.24, 0.37]
Age						
Age (continuous)	−0.00	−0.011	0.01	−0.28	0.783	[−0.01, 0.01]
Migration background						
Migration background (ref.: no migration background)	−0.04	−0.006	0.25	−0.17	0.863	[−0.54, 0.45]
Education						
Vocational training (ref.: no vocational training)	0.07	0.014	0.23	0.30	0.776	[−0.39, 0.53]
University degree (ref.: no vocational training)	0.87	0.148	0.27	3.20	0.001	[0.34, 1.40]
Employment status						
Employed (ref.: unemployed)	0.21	0.044	0.18	1.17	0.241	[−0.14, 0.56]
Area						
Urban (ref.: rural)	−0.17	−0.036	0.16	−1.10	0.273	[−0.48, 0.14]
Media exposure						
Media exposure	0.00	0.000	0.08	0.000	0.997	[−0.16, 0.16]
Constant						
Constant	14.08		0.56	25.28	<0.001	[12.99, 15.18]

*Ref. = reference group*.

a Age groups: group 1 (18–30), group 2 (31–40), group 3 (41–50), group 4 (51–60), group 5 (61–70), group 6 (>70).

b The value of media width varies between 0 (broad media use) and 1 (one-sided media use).

### Perceived Knowledge Hypothesis

For the perceived knowledge hypothesis, we conducted separate analyses (regression analyses and dose–response functions) for media exposure and media width. For the regression of perceived knowledge on media exposure, the regression equation referring to the entire model (see Table 5a), was significant, *F*(14,937) = 16.51, *p* < 0.001, *R*^2^ = 0.198. When controlling for all covariates, media exposure was positively linked to perceived knowledge about COVID-19, *b* = 0.47, *p* < 0.001. The additional variance explained by the predictor media exposure was *ΔR*^2^ = 0.130. We further estimated the dose–response function based on the GPS for media exposure. The media exposure was normally distributed at level *p* = 0.05. The balancing property was satisfied at level *p* = 0.20, i.e., evidence supports the balancing property. This analysis also supported the results of the regression analysis. Media exposure was positively associated with perceived knowledge about COVID-19, *b* = 0.47, *p* < 0.001, i.e., the higher the media exposure, the higher the perceived knowledge about COVID-19.

**Table 5A:** Regression of perceived knowledge on media exposure.

Variable	*B*	*β*	*SE*	*t*	*p*	95% *CI*
Media exposure						
Media exposure	0.47	0.464	0.04	12.30	<0.001	[0.40, 0.55]
Perceived threat						
Perceived threat	0.05	0.065	0.02	2.07	0.039	[0.00, 0.10]
Gender						
Male (ref.: female)	−0.01	−0.006	0.07	−0.19	0.850	[−0.15, 0.13]
Age group^a^						
2 (ref.: group 1)	−0.23	−0.065	0.13	−1.80	0.073	[−0.47, 0.02]
3 (ref.: group 1)	0.04	0.012	0.13	0.33	0.744	[−0.21, 0.29]
4 (ref.: group 1)	0.14	0.048	0.12	1.20	0.231	[−0.09, 0.38]
5 (ref.: group 1)	0.03	0.010	0.13	0.22	0.829	[−0.22, 0.28]
6 (ref.: group 1)	0.19	0.044	0.17	1.12	0.263	[−0.14, 0.53]
Migration background						
Migration background (ref.: no migration background)	−0.12	−0.031	0.12	−1.02	0.307	[−0.35, 0.11]
Education						
Vocational training (ref.: no vocational training)	0.10	0.039	0.11	0.90	0.371	[−0.12, 0.31]
University degree (ref.: no vocational training)	0.04	0.013	0.13	0.31	0.755	[−0.21, 0.29]
Employment status						
Employed (ref.: unemployed)	0.07	0.029	0.09	0.80	0.426	[−0.10, 0.25]
Area						
Urban (ref.: rural)	0.21	0.085	0.07	2.86	0.004	[0.07, 0.35]
Media width^b^						
Media width^b^	2.16	0.219	0.36	5.94	<0.001	[1.44, 2.87]
Constant						
Constant	2.47		0.25	9.89	<0.001	[1.98, 2.96]

*Ref. = reference group*.

a *Age groups: group 1 (18–30), group 2 (31–40), group 3 (41–50), group 4 (51–60), group 5 (61–70), group 6 (>70)*.

b *The value of media width varies between 0 (broad media use) and 1 (one-sided media use)*.

Regarding media width, the significant regression equation, referring to the entire model, *F*(10,941) = 22.14, *p* < 0.001, *R*^2^ = 0.191, is displayed in Table 5b. The results revealed that while controlling for all covariates, the standardized Lorenz coefficient was positively linked to perceived knowledge about COVID-19, *b* = 2.21, *p* < 0.001. The additional variance explained by the predictor media width was *ΔR*^2^ = 0.032. Again, we estimated dose–response functions based on the GPS for the standardized Lorenz coefficient. The balancing property was satisfied at level *p* = 0.10, indicating only very slight evidence against the balancing property. This analysis revealed similar results. In particular, a lower width of media use (i.e., a higher Lorenz coefficient) was associated with higher perceived knowledge, *b* = 2.11, *p* < 0.001.

**Table 5B:** Regression of perceived knowledge on media width.

Variable	*B*	*β*	*SE*	*t*	*p*	95% *CI*
Media width^b^						
Media width^b^	2.21	0.225	0.36	6.09	<0.001	[1.50, 2.93]
Perceived threat						
Perceived threat	0.05	0.066	0.02	2.13	0.034	[0.00, 0.10]
Gender						
Male (ref.: female)	−0.01	−0.002	0.07	−0.07	0.941	[−0.15, 0.14]
Age						
Age (continuous)	0.00	0.041	0.00	1.11	0.268	[−0.00, 0.01]
Migration background						
Migration background (ref.: no migration background)	−0.14	−0.035	0.12	−1.15	0.250	[−0.37, 0.10]
Education						
Vocational training (ref.: no vocational training)	0.10	0.039	0.11	0.90	0.367	[−0.12, 0.31]
University degree (ref.: no vocational training)	0.03	0.011	0.13	0.25	0.799	[−0.22, 0.28]
Employment status						
Employed (ref.: unemployed)	0.05	0.021	0.08	0.60	0.549	[−0.11, 0.21]
Area						
Urban (ref.: rural)	0.21	0.084	0.07	2.83	0.005	[0.06, 0.35]
Media exposure						
Media exposure	0.47	0.463	0.04	12.31	<0.001	[0.40, 0.55]
Constant						
Constant	2.34		0.26	9.02	<0.001	[1.83, 2.85]

*Ref. = reference group*.

a Age groups: group 1 (18–30), group 2 (31–40), group 3 (41–50), group 4 (51–60), group 5 (61–70), group 6 (>70).

b The value of media width varies between 0 (broad media use) and 1 (one-sided media use).

### Exploratory Analyses – The Effect of Different Media Channels

For our exploratory analyses regarding the associations between the usage of different media channels and socio-demographic characteristics with actual and perceived knowledge about COVID-19, we conducted regression analyses (see Tables 6a,6b).

**Table 6A:** Regression of actual knowledge.

Variable	*B*	*β*	*SE*	*t*	*p*	95% *CI*
Media channel						
Public television	−0.01	−0.006	0.05	−0.15	0.880	[−0.11, 0.09]
Private television	−0.06	−0.052	0.04	−1.36	0.174	[−0.14, 0.03]
Newspapers	−0.03	−0.025	0.04	−0.71	0.481	[−0.10, 0.05]
Online news	0.25	0.224	0.04	6.26	<0.001	[0.17, 0.33]
Radio	−0.05	−0.044	0.04	−1.27	0.204	[−0.12, 0.03]
Social Media	−0.22	−0.215	0.04	−5.46	<0.001	[−0.30, −0.14]
Podcasts	0.06	0.043	0.05	1.19	0.233	[−0.04, 0.17]
Age						
Age (continuous)	0.00	0.000	0.01	0.03	0.996	[−0.01, 0.01]
Gender						
Male (ref.: female)	−0.01	−0.003	0.15	−0.10	0.921	[−0.31, 0.28]
Migration background						
Migration background (ref.: no migration background)	−0.05	−0.007	0.25	−0.21	0.833	[−0.54, 0.43]
Education						
Vocational training (ref.: no vocational training)	−0.01	−0.003	0.23	−0.06	0.953	[−0.46, 0.43]
University degree (ref.: no vocational training)	0.59	0.100	0.27	2.19	0.029	[0.06, 1.11]
Employment status						
Employed (ref.: unemployed)	0.15	0.032	0.18	0.87	0.385	[−0.19, 0.50]
Area						
Urban (ref.: rural)	−0.15	−0.032	0.15	−0.99	0.320	[−0.46, 0.15]
Constant						
Constant	15.05		0.41	36.57	<0.001	[14.24, 15.86]

*Ref. = reference group*.

**Table 6B:** Regression of perceived knowledge.

Variable	*B*	*β*	*SE*	*t*	*p*	95% *CI*
Media channel						
Public television	0.12	0.198	0.02	5.05	<0.001	[0.08, 0.17]
Private television	0.03	0.043	0.02	1.20	0.232	[−0.02, 0.07]
Newspapers	0.05	0.096	0.02	2.80	0.005	[0.02, 0.09]
Online news	0.09	0.156	0.02	4.56	<0.001	[0.05, 0.13]
Radio	0.02	0.045	0.02	1.33	0.182	[−0.01, 0.06]
Social Media	0.04	0.084	0.02	2.24	0.025	[0.01, 0.08]
Podcasts	0.00	0.004	0.03	0.11	0.911	[−0.05, 0.05]
Age						
Age (continuous)	0.00	0.058	0.00	1.44	0.152	[−0.00, 0.01]
Gender						
Male (ref.: female)	−0.02	−0.008	0.07	−0.27	0.786	[−0.16, 0.12]
Migration background						
Migration background (ref.: no migration background)	−0.13	−0.035	0.12	−1.12	0.262	[−0.37, 0.10]
Education						
Vocational training (ref.: no vocational training)	0.08	0.031	0.11	0.70	0.485	[−0.14, 0.30]
University degree (ref.: no vocational training)	−0.03	−0.009	0.13	−0.20	0.839	[−0.28, 0.23]
Employment status						
Employed (ref.: unemployed)	0.05	0.022	0.09	0.63	0.532	[−0.11, 0.22]
Area						
Urban (ref.: rural)	0.20	0.082	0.07	2.70	0.007	[0.05, 0.35]
Constant						
Constant	3.48		0.20	17.38	<0.001	[3.09, 3.87]

*Ref. = reference group*.

For actual knowledge, the significant regression equation referring to the entire model, *F*(14,937) = 6.16, *p* < 0.001, *R*^2^ = 0.084, revealed a positive effect of online news use (*b* = 0.25, *p* < 0.001), whereas social media use to inform oneself about COVID-19 showed a negative effect (*b* = −0.22, *p* < 0.001) while controlling for all covariates. For perceived knowledge, the significant regression equation referring to the entire model, *F*(14,937) = 13.52, *p* < 0.001, *R*^2^ = 0.168, revealed positive effects for using public television (*b* = 0.12, *p* < 0.001), online news (*b* = 0.09, *p* < 0.001), newspapers (*b* = 0.05, *p* = 0.005), and social media (*b* = 0.04, *p* = 0.025) when controlling for all covariates. Taken together, corresponding associations for both actual and perceived knowledge only emerged for using online news. In contrast, social media use was positively linked to perceived knowledge, whereas it was negatively linked to actual knowledge. Public television use and newspapers use was associated with perceived knowledge, without showing associations with actual knowledge.

Regarding the socio-demographic variables entered in the regression, the results revealed a positive effect of education on actual knowledge about COVID-19, with a university degree showing higher actual knowledge compared to having no vocational training (*b* = 0.59, *p* = 0.029). Further, living in an urban area was related to higher perceived knowledge than living in a rural area (*b* = 0.20, *p* = 0.007). For age, gender, migration background, and employment status, no effects were revealed.

### Exploratory Analyses – Mediation

To explore whether an association emerged between perceived threat and both perceived and actual knowledge and whether this association was mediated by participants’ media use, we conducted mediation analyses using the PROCESS macro in SPSS (Hayes, 2017).

First, we tested whether there was an association between perceived threat and perceived knowledge and whether this association was mediated by media exposure or media width, respectively. Analyses showed a significant total effect of perceived threat on perceived knowledge, *b* = 0.15, *p* < 0.001 [CI_95%_: 0.10, 0.20]. In the mediation model including media exposure as a mediator, there was a significant effect of threat on media exposure, *b* = 0.22, *p* < 0.001 [CI_95%_: 0.18, 0.27] as well as a significant effect of media exposure on perceived knowledge, *b* = 0.34, *p* < 0.001 [CI_95%_: 0.28, 0.41]. Further, after adding media exposure as a mediator, there still was a direct effect of threat on perceived knowledge, *b* = 0.07, *p* = 0.003 [CI_95%_: 0.02, 0.12], which was somewhat smaller than the total effect not including the mediator. Because the confidence interval for the indirect effect did not include 0, this partial mediation can be considered significant, *b* = 0.08 [CI_95%_: 0.06, 0.10]. The model including media width as a mediator revealed no mediation because the confidence interval for the indirect effect included 0, *b* = 0.001 [CI_95%_: −0.002, 0.006].

With regard to the association of perceived threat and actual knowledge, the analyses revealed no total effect of threat on actual knowledge, *b* = 0.00, *p* = 0.941 [CI_95%_: −0.09, 0.10]. Neither of the two mediation models revealed indirect effects for media exposure, *b* = −0.02 [CI_95%_: −0.05, 0.01] or media width, *b* = −0.01 [CI_95%_: −0.02, 0.001], respectively. Hence, there were no indications for media width as a mediator.

## Discussion

The COVID-19 pandemic is a global health crisis, and its successful containment depends on people’s actual knowledge about the virus. In this highly dynamic global situation, media play an important role in communicating knowledge about the pandemic to the public. The reported study was the first to investigate systematic relationships between perceived threat and media use as well as between media use and pandemic-related knowledge. Given that the online study was conducted within the first months of the COVID-19 pandemic (April 2020), we were able to capture the parameters of interest in a crucial time of the novel situation, when public knowledge about the pandemic was not yet very developed.

The results provide evidence for the threat hypothesis assuming that higher levels of perceived threat were associated with higher media exposure. Hence, in line with prior research on information seeking during crises (e.g., Spence et al., 2005; Lachlan et al., 2009), the results support the assumptions of the uncertainty reduction theory (Berger and Calabrese, 1975) and media system dependency theory (Ball-Rokeach and DeFleur, 1976). In particular, individuals who felt more threatened by COVID-19 used media more frequently (i.e., higher media exposure) to meet their need for information. However, since our analyses rely on cross-sectional data that do not allow for causal inferences, an explanation that assumes a different direction might also be conceivable: For example, high exposure to media might increase one’s perceptions of threat due to COVID-19 (see cultivation theory, Gerbner, 1998). Interestingly, regarding media width, the results showed that contrary to our hypothesis, a higher perceived threat was linked to a smaller media width. Hence, individuals who reported higher levels of perceived threat tended to limit their media use to fewer media channels. Integrating both of these findings, it appears that with higher levels of perceived threat, individuals used media channels rather more deeply than broadly: People tended to focus on fewer media channels, which they used intensely.

Regarding media exposure, the results do not support the actual knowledge hypothesis regarding media exposure and prior research results (De Vreese and Boomgaarden, 2006; Kaufhold et al., 2010; Marquart et al., 2019), since increased media exposure was not associated with one’s actual knowledge about the COVID-19 pandemic. However, based on prior research (e.g., Zhao, 2009), and in support of our perceived knowledge hypothesis for media exposure, we found an association between media exposure and perceived knowledge: The higher one’s media exposure, the higher one’s perceived knowledge about COVID-19. Taken together, the results suggest that with increased media exposure, individuals think that they are better informed without showing higher levels of actual knowledge. Hence, with increased media exposure, we observed an illusion of knowledge, which has already been documented in previous studies (Leonhard et al., 2020; Schäfer, 2020). Repeated exposure to information about COVID-19 may have induced overconfidence in one’s knowledge (Koriat, 1995; Kardas and O’Brien, 2018).

For media width, contrary to the actual knowledge hypothesis, we found that a lower media width was associated with higher levels of actual knowledge. This association was also found between media width and perceived knowledge. Individuals, who focused their media use on fewer different media channels, felt better informed and were better informed about COVID-19. As one possible explanation regarding the effects on actual knowledge, it is conceivable that a more focused media use reflects stronger elaboration within one media channel instead of using different media channels rather superficially. However, further research is needed to corroborate this tentative explanation. Regarding perceived knowledge, a higher media width may increase the probability of consuming different or even inconsistent information. Therewith, individuals may become aware of their knowledge gaps regarding the pandemic, which in turn results in lower levels of perceived knowledge.

Regarding our exploratory analyses addressing the associations between the usage of different media channels with actual and perceived knowledge about COVID-19, we observed an illusion of knowledge for using public television, newspapers, and social media (also see Schäfer, 2020). Interestingly, we found social media use to be linked to higher perceived knowledge scores, while it was actually linked to lower actual knowledge scores. A potential explanation for this reversed relationship may be that on social media, individuals can share and receive any information without content verification and validation. This suggests that social media as a source of information should be treated with high caution by users. Regarding our exploratory mediation analyses, the results suggest that the relationship between perceived threat and perceived knowledge can be partially explained by media exposure.

Taken together, this study takes a first step in describing and understanding how the perceived threat is associated with media use (i.e., media exposure, media width) and how these patterns are linked to perceived and actual knowledge about the pandemic. The findings suggest that people who are well informed about the COVID-19 pandemic appear to consume media in a more focused manner (fewer channels).

### Limitations and Directions for Future Research

When interpreting the results of this study, different limitations should be considered. These limitations mainly emerge from unobserved heterogeneity that we could not control in a setting that tried to tackle the research gaps in a dynamic real-world pandemic situation. However, since we only collected cross-sectional data, we aimed at accounting for observed and unobserved heterogeneity by applying restrictive analyses. Moreover, given that we found small effects, there is still unexplained variance that should be addressed in future research. Further, the reliability of our knowledge test on COVID-19 was comparatively low. However, this low internal consistency may be due to the heterogeneity of knowledge about COVID-19. Consequently, maximizing homogeneity and thus internal consistency may result in constructs being operationalized too narrowly (see Stadler et al., 2021 for an in-depth discussion of this issue). It should also be noted that in this study, we did not consider the differences within the media channels since we only accounted for the global media use of a specific media channel. Therefore, future research should also address how the investigated variables are associated with different formats within the channels. Additionally, given that the study was conducted within the first months of the COVID-19 pandemic, it is conceivable that certain mechanisms (such as the identified knowledge illusions) have changed over the course of the pandemic. Therefore, future research should address whether the results also hold true for later phases of the pandemic to determine whether such mechanisms are stable over time. These studies could also investigate whether persistent illusions of knowledge are related to conspiracy beliefs and susceptibility to false information about the pandemic. In this regard, previous research in other contexts already identified associations between illusions of understanding and the endorsement of conspiracy beliefs (Vitriol and Marsh, 2018), as well as political extremism (Fernbach et al., 2013).

### Implications

Our results suggest that illusions of knowledge are more pronounced for heavy users of social media. Whereas we did not collect any data on potential mechanisms underlying this observation, it depicts a promising pathway for further research that investigates whether this observation is driven by the problematic distribution of misinformation on social networking sites (Vosoughi et al., 2018) or by an oversimplification of information, which may result in illusions of understanding (see Scharrer et al., 2017). In this regard, future studies could examine the impact of different design features on knowledge acquisition. Based on these studies, it could be possible to derive recommendations on how to communicate scientific information (especially relevant for media organizations and health officials) and evaluate information on the internet (especially relevant for users). In terms of communication strategies, it may be necessary to consider the trade-off between comprehensibility of information and oversimplification. With regard to the evaluation of information, it may be necessary to teach users how to evaluate the credibility of information on the internet (see Anmarkrud et al., 2021 for an overview of research on source evaluation). Based on the results of our study, it may be advisable to rely on information from more traditional online news instead of information from social media because there was a positive association between online news use and both actual and perceived knowledge, respectively. In contrast, there were indications for illusions of knowledge for social media use.

### Conclusion

This study contributes to the literature as one of the first studies that investigates the interplay of perceived threat, media use, and perceived and actual knowledge in the context of the COVID-19 pandemic. The results point to different key messages: First, people who feel more threatened by COVID-19 consume media more often but focus on fewer different media channels. Second, individuals who frequently use media to inform themselves about the pandemic feel better informed without actually gaining more knowledge about COVID-19 (i.e., illusion of knowledge). Third, individuals who consume media in a more focused way (i.e., lower media width) tend to feel and be better informed about COVID-19. Exploratory analyses for the different media channels also point to illusions of knowledge for public television, newspapers, and especially for social media.

Taken together, the present study takes a first step at understanding how knowledge about COVID-19 may be built by using media and how using media may contribute to illusions of knowledge. Given that overcoming crises such as the COVID-19 pandemic depends on profound crisis-related knowledge within the general population, the current study represents a starting point for future research on the role of media for acquiring knowledge about global crises, also beyond COVID-19 (e.g., climate change).

## Data Availability Statement

The datasets presented in this study can be found in online repositories. The names of the repository/repositories and accession number(s) can be found at: https://osf.io/869t3/?view_only=564785998bc443488ea9d69e8f3bc961.

## Ethics Statement

The studies involving human participants were reviewed and approved by Local Ethics Committee (ethikkommission@die-bonn.de) at the German Institute for Adult Education - Leibniz Centre for Lifelong Learning (approval number: DIE-LEK 2020-002). The patients/participants provided their written informed consent to participate in this study.

## Author Contributions

JG and CS: writing - original draft and project administration. JG, CS, MM, and AM: writing - reviewing and editing, conceptualization, methodology, and data analysis. AM and MM: supervision. All authors contributed to the article and approved the submitted version.

## Conflict of Interest

The authors declare that the research was conducted in the absence of any commercial or financial relationships that could be construed as a potential conflict of interest.

## Publisher’s Note

All claims expressed in this article are solely those of the authors and do not necessarily represent those of their affiliated organizations, or those of the publisher, the editors and the reviewers. Any product that may be evaluated in this article, or claim that may be made by its manufacturer, is not guaranteed or endorsed by the publisher.

## Supplementary Material

The Supplementary Material for this article can be found online at: https://www.frontiersin.org/articles/10.3389/fpsyg.2021.778886/full#supplementary-material

## Footnotes

^1^For example, participants were excluded who stated that they frequently used podcasts to inform themselves about COVID-19 but noted in a later question that they had never heard a podcast.

^2^At the beginning of the survey, we informed the participants that we used the common term “coronavirus” instead of the scientific term for better understanding.

^3^Please note that the data reported in this manuscript constitutes a subset of the data collected that covered different aspects of media use during the COVID-19 pandemic. The full dataset, including all variables, can be downloaded at OSF (https://osf.io/869t3/?view_only=564785998bc443488ea9d69e8f3bc961).

^4^Given that our constructs of interest (i.e., perceived threat, media exposure, and media width) are continuous variables, conventional propensity score matching building on binary treatments was not applicable.

^5^ https://osf.io/869t3/?view_only=564785998bc443488ea9d69e8f3bc961

^6^We calculated *Δ*^2^ using stepwise regression analyses in which we entered all variables except the variable of interest in a first step and the variable of interest in a second step. To keep the results section short and precise, we decided not to report all the stepwise analyses and limit reporting to the *Δ*^2^ value.
