# Supplementary material for: Actual and Perceived Knowledge About COVID-19: The Role of Information Behavior in Media
Source: Front Psychol. 2021 Dec 15;12:778886. doi: 10.3389/fpsyg.2021.778886 (PMC8714891; doi:10.3389/fpsyg.2021.778886)
Supplement: Supplementary file 1 [file Data_Sheet_1.PDF]

Table A.1

Knowledge Test on Actual Knowledge about COVID-19 (True/False Statements)

	Item	Correct answer	Correct rate (in %)
1	The correct name for the disease caused by the coronavirus is "SARS-CoV-2".	false	35.61%
2	According to the current state of research, scientists assume that the transmission of the coronavirus originated at a wild animal market in Wuhan.	true	85.82%
3	COVID-19 stands for "COronaVirus Infectious Disease type E23119".	false	47.37%
4	The WHO currently warns against taking Ibuprofen if corona disease is suspected.	false	59.87%
5	According to current research, 70% of corona diseases in China were mild to moderate, 15% were severe but not life-threatening and 15% were critical to life-threatening.	false	22.79%
6	According to current research, fever and cough are among the most common symptoms of a corona disease.	true	96.01%
7	According to the current state of research, people with diseases of the cardiovascular system are among the groups of people with an increased risk of severe courses.	true	88.24%
8	The term "reproduction number" describes the duration of the doubling of the cases (in days).	false	23.32%
9	According to the current state of research, the main transmission route of the coronavirus is by droplet infection.	true	94.33%
10	Widely referred to as the "coronavirus", the virus is scientifically referred to as "COVID-19".	false	5.99%
11	According to the Robert Koch Institute, ‘category I contact persons with close contact’ (higher risk of infection) are persons who had face-to-face contact with an infected person for at least 30 minutes.	false	41.07%
12	According to the current state of research, there is growing evidence that bats were involved in the transmission of the coronavirus.	true	72.58%
13	The current coronavirus rapid test is performed using the PCR method (polymerase chain reaction).	true	65.23%
14	Corona viruses belong to the family of DNA viruses.	false	70.06%

15	The term "zoonosis" describes diseases that are naturally transmissible from animals to humans and from humans to animals.	true	59.77%
16	The term "virulence" describes the pathogenic potential of a pathogen.	true	65.65%
17	Scientists refer to "patient 1" as the first person to fall ill, from whom the spread of a disease started.	false	18.17%
18	According to current research, rhinitis, diarrhoea and headaches are rare symptoms of corona disease.	true	63.55%
19	Both the Robert Koch Institute and the Johns Hopkins University publish information on current infection and death rates, although the rates of the Johns Hopkins University are usually somewhat lower than those of the Robert Koch Institute.	false	39.60%
20	According to current research, the incubation period of the coronavirus is on average 14 days.	false	9.98%
21	A flu vaccination does not prevent infection with the coronavirus.	true	89.50%
22	By testing for antibodies against the coronavirus, it can be determined whether the person being tested has already been infected.	true	89.08%
23	The abbreviation for the novel coronavirus SARS-CoV 2 stands for "Severe Acute Respiratory Syndrome Coronavirus-2".	true	65.86%
24	The drug that was developed against Ebola viruses and is now being used in studies on the therapy of corona patients with particularly severe courses is called ‘Remostonin’.	false	45.59%
25	The term "incubation period" describes the time between infection and the first day on which symptoms appear.	true	90.97%
26	According to the current state of research, pregnant women are among the groups of persons with an increased risk of severe courses of disease.	false	60.08%

Note. The answer options were “true” and “false”. The items were translated to English (original: German). The time limit was 30 seconds per item.
